# Disrupted astrocyte-neuron metabolic coupling in amyotrophic lateral sclerosis

**DOI:** 10.3389/fcell.2026.1899307

**Published:** 2026-08-20

**Authors:** Áine Bríd Heffernan, Florence Do, Eleanor Carter, Farah Al-Tameemi, Bhuvaneish T. Selvaraj, Maria Stavrou

**Affiliations:** 1 UK Dementia Research Institute at University of Edinburgh, University of Edinburgh, Edinburgh, United Kingdom; 2 Institute for Neuroscience and Cardiovascular Research, University of Edinburgh, Edinburgh, United Kingdom; 3 Euan MacDonald Centre for MND Research, University of Edinburgh, Edinburgh, United Kingdom; 4 Edinburgh Medical School, The University of Edinburgh, Edinburgh, United Kingdom; 5 Anne Rowling Regenerative Neurology Clinic, University of Edinburgh, Edinburgh, United Kingdom

**Keywords:** amyotrophic lateral sclerosis, astrocyte, neuron, metabolism, bioenergetics, cell crosstalk

## Abstract

Amyotrophic lateral sclerosis (ALS) is a fatal neurodegenerative disease characterised by progressive loss of motor neurons. In addition to neurodegeneration, ALS is increasingly recognised as a disorder associated with widespread metabolic dysfunction, including hypermetabolism, weight loss, and dyslipidaemia, all of which correlate with disease progression and survival. Astrocytes play a central role in maintaining metabolic homeostasis in the central nervous system by supporting neuronal energy demands, regulating glutamate levels, buffering oxidative stress, and maintaining lipid balance. Emerging evidence suggests that disruption of these supportive astrocytic functions may contribute directly to motor neuron vulnerability in ALS. In this mini-review, we discuss how alterations in astrocyte metabolism may impair astrocyte-neuron metabolic coupling in ALS. We summarise work from human studies and experimental models demonstrating abnormalities in astrocytic glycolysis, mitochondrial function, lactate shuttling, lipid metabolism, and glutamate homeostasis. We highlight growing evidence implicating mitochondrial dysfunction and impaired lipid handling in astrocytes as important contributors to disease progression. We explore how these changes may deprive motor neurons of metabolic and antioxidant support while also promoting excitotoxicity, oxidative stress, and lipotoxicity. We also discuss how recent advances in human induced pluripotent stem cell models, metabolomics, and single-cell transcriptomics are improving our understanding of astrocyte dysfunction in ALS. Finally, we consider current and emerging therapeutic strategies aimed at restoring astrocytic metabolic function. Together, these findings support the idea that progressive failure of astrocyte-mediated metabolic support is an important component of ALS pathogenesis and may represent a promising therapeutic target.

## 1 Introduction

Amyotrophic lateral sclerosis (ALS) is a debilitating and fatal neurodegenerative disease with neither cure nor effective disease modifying treatments. Although it is characterised by selective loss of motor neurons (MNs) in the motor cortex and spinal cord, accumulating evidence implicates astrocytes as important non-cell autonomous contributors to ALS pathogenesis. Cytoplasmic aggregation of TDP-43 is a neuropathological hallmark, observed in c.97% of all ALS cases, including both sporadic and genetic forms of disease (Mackenzie et al., 2010). While most ALS cases are sporadic (sALS), familial genetic mutations account for c.10% of cases, with genetic studies identifying a plethora of genes linked to ALS including *C9ORF72*, *SOD1*, *FUS* and *TARDBP* (Hardiman et al., 2017).

Nevertheless, familial and sporadic forms of ALS are clinically indistinguishable, with systemic metabolic dysfunction including weight loss, hypermetabolism and dyslipidaemia displayed across all patients (Vandoorne et al., 2018). ALS patients often demonstrate hypermetabolism with muscle atrophy and weight loss before loss of MNs and onset of motor symptoms, demonstrating the pivotal role of metabolic changes in the early stages of disease development (Peter et al., 2017; Dorst et al., 2023). These metabolic changes correlate with disease prognosis and survival, as hypermetabolic ALS patients have a faster rate of functional decline and shorter survival, underscoring the key role that metabolic dysfunction plays in ALS (Steyn et al., 2018; Zanovello et al., 2022). This is also reflected at a cellular and molecular level as mitochondrial dysfunction and dyslipiemia are commonly observed.

Given the central role of astrocytes in maintaining central nervous system (CNS) metabolic homeostasis, astrocytic dysfunction may represent a key cellular link between systemic metabolic abnormalities and MNs degeneration in ALS. Here, we outline emerging evidence from experimental models and human studies which suggests that disruptions in astrocytic bioenergetics, including abnormalities in glycolysis, mitochondrial function, lactate shuttling, lipid metabolism, and glutamate homeostasis, play a key role in ALS pathogenesis. We then discuss how these impairments to astrocytic metabolic homeostasis may contribute to MN vulnerability. Finally we explore the translational potential of targeted astrocytic metabolic strategies which offer an alternative, promising approach in the development of new treatments for ALS.

## 2 Deficits in astrocytic bioenergetics in ALS

**FIGURE 1:**
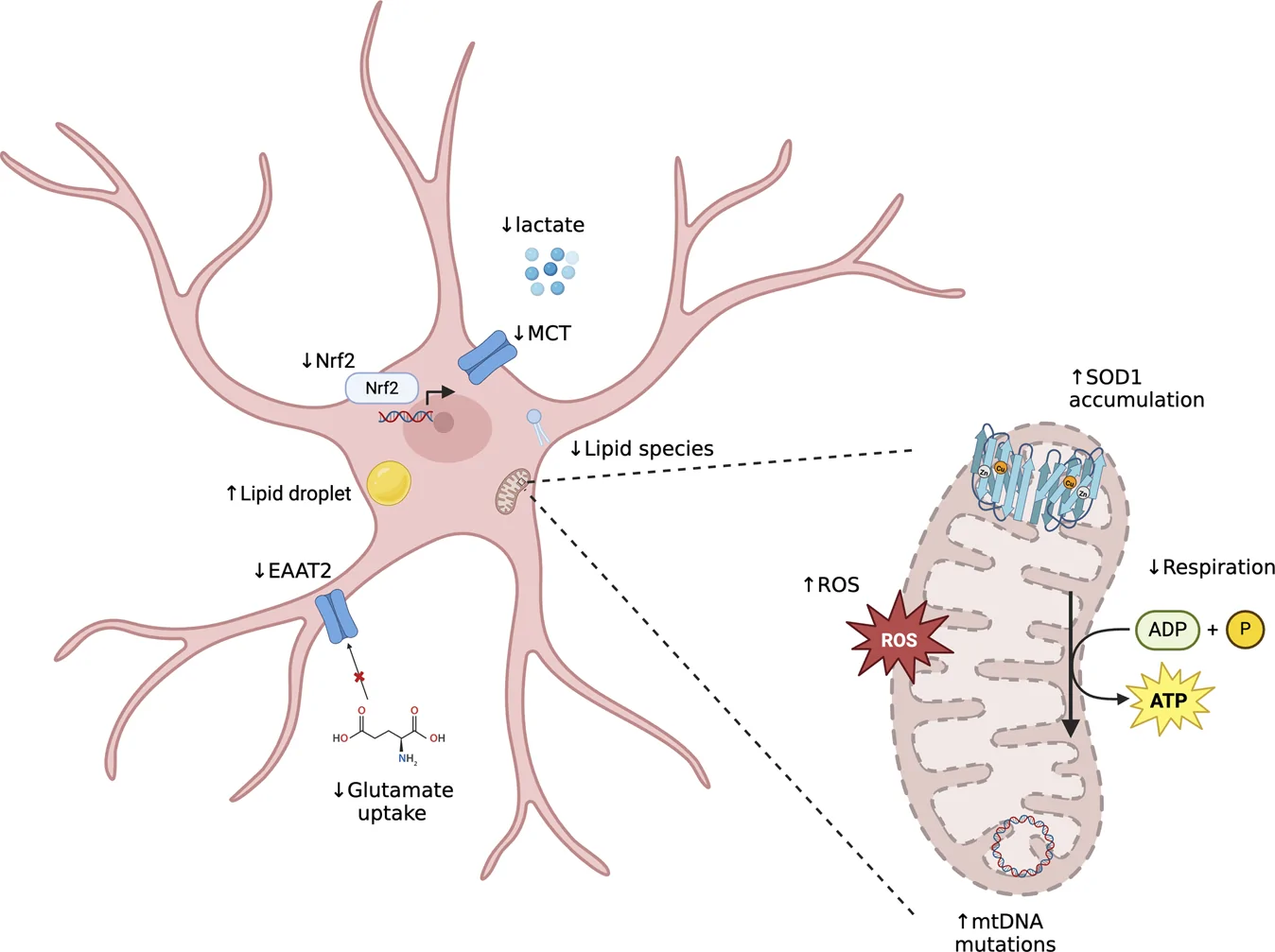
Bioenergetic dysfunction in ALS astrocytes. Astrocytes play a critical role as metabolic regulators in the brain. In ALS, they undergo metabolic reprogramming which is characterised by impaired glutamate clearance, reduced lactate availability, lipid droplet accumulation, decreased antioxidant capacity and mitochondrial dysfunction. ALS, amyotrophic lateral sclerosis; EAAT2, excitatory amino acid transporter 2; MCT, monocarboxylate transporter; mtDNA, mitochondrial DNA; Nrf2, nuclear factor erythroid 2-related factor 2; ROS, reactive oxygen species. Figure created with BioRender.com.

Astrocytes play a central role in maintaining metabolic homeostasis in the CNS by supporting neuronal energy demands, regulating glutamate levels, buffering oxidative stress, and maintaining lipid balance. Across multiple neurological conditions, astrocytes undergo profound metabolic remodelling as part of a dynamic cellular response to CNS injury and damage (Polyzos et al., 2019; Le Douce et al., 2020). In ALS, emerging evidence suggests that astrocytes adopt a metabolically altered state characterised by impaired substrate utilisation, mitochondrial dysfunction, and reduced capacity to sustain neuronal metabolic support under stress (Figure 1). This phenotype is likely shaped not only by intrinsic bioenergetic deficits but also by the inflammatory milieu of the diseased CNS. Indeed, inflammatory signalling has been shown to remodel astrocytic metabolism, altering mitochondrial activity and oxidative metabolism while promoting reactive astrocyte states (Motori et al., 2013; Chao et al., 2019). Thus, neuroinflammation and bioenergetic deficits are inextricably linked and their synergistic contribution to disease progression should be considered.

### 2.1 Astrocytic metabolic dysfunction

Astrocytes and neurons possess distinct metabolic profiles and co-operate via complex metabolic networks for optimal, efficient function. Astrocytes preferentially undergo glycolysis, utilising glucose and mobilising glycogen stores during periods of high neuronal activity, whilst neurons heavily rely on mitochondrial oxidative phosphorylation (OXPHOS) and utilise glucose-derived pyruvate and astrocyte-derived lactate to support adenosine triphosphate (ATP) production (Supplie et al., 2017). The astrocyte-neuron lactate shuttle (ANLS) describes this division of labour: glutamate uptake at the synapse triggers aerobic glycolysis and lactate secretion by astrocytes, which neurons import via monocarboxylate transporters (MCT) for oxidative ATP production (Magistretti and Allaman, 2015). Most importantly, lactate is not merely an alternative substrate but directly mediates the effect of glucose on neuronal depolarisation (Sada et al., 2015) and rescues synaptic plasticity impaired by stress (Murphy-Royal et al., 2020), providing strong support for the ANLS and its neuroprotective effect.

Altered glucose utilisation is observed in ALS, as captured in patients by ^18^F-fluorodeoxyglucose PET (FDG-PET) scans, with C9ORF72 and sALS patients showing hypometabolism when compared to neurologically normal controls (Dalakas et al., 1987; Cistaro et al., 2014; Di Pede et al., 2026). However, regions such as the brainstem and cervical cord of ALS patients have been shown to display hypermetabolism (Zanovello et al., 2022). These opposing findings likely reflect regional and temporal heterogeneity, with neuronal hypometabolism co-existing alongside inflammatory-driven glial hypermetabolism.

Reduced lactate levels have been observed in astrocytic culture medium (Ferraiuolo et al., 2011; Madji Hounoum et al., 2017), suggestive of metabolic dysfunction. The reduced expression of lactate transporters further limits lactate availability to MNs, leaving them in energetic deprivation (Ferraiuolo et al., 2011). This aligns with clinical data where lower serum lactate levels correlates with shorter survival in ALS patients (Nakamura et al., 2026), supporting an association between impaired metabolic flexibility and disease progression.

Central to the astrocytic metabolic shift observed in ALS is a collapse of mitochondrial bioenergetics with reduced mitochondrial respiration observed in SOD1-G93A rodent astrocytes (Cassina et al., 2008; Miquel et al., 2012; Martínez-Palma et al., 2019). Increased burden of mitochondrial DNA (mtDNA) mutations may contribute to this reduction in OXPHOS, as analysis of C9ORF72 cerebral organoids revealed preferential accumulation of mtDNA mutations in astroglia (Nie et al., 2025). Structural integrity of astrocytic mitochondria may be further compromised by the accumulation of mutant proteins such as superoxide dismutase 1 (SOD1) (Vande Velde et al., 2008) and a significant loss of cardiolipin, essential for cristae morphology and ATP production (Chaves-Filho et al., 2019). This reduced respiratory capacity may initiate a self-perpetuating cycle of oxidative stress. Increased production of reactive species such as peroxynitrite can then further exacerbate mitochondrial dysfunction. Additionally, C9ORF72-mutant astrocytes demonstrate a loss of metabolic flexibility under starvation, involving defects in glycogen and adenosine metabolism (Allen et al., 2019). Together, these findings suggest that impaired substrate utilisation and mitochondrial dysfunction converge to reduce astrocyte-mediated energetic support in ALS.

### 2.2 Lipid metabolism dysregulation

Beyond glucose metabolism, astrocytes are also key players in lipid metabolism. Most ALS patients exhibit hypolipidemia, characterised by reduced serum cholesterol, low-density lipoprotein (LDL), and triglyceride levels (Yang et al., 2013). Conversely, elevated serum cholesterol and triglyceride levels have been associated with prolonged survival (Janse van Mantgem et al., 2023; Dorst et al., 2011). Epidemiological studies suggest that the consumption of foods rich in polyunsaturated fatty acids (PUFA) may delay the onset of ALS (Fitzgerald et al., 2014). Furthermore, genome-wide DNA methylation studies have identified differentially methylated positions in genes associated with cholesterol biosynthesis pathways (Hop et al., 2022). Recent Mendelian randomisation identified longitudinal changes in lipid and carnitine metabolism in plasma and postmortem tissue which correlated with ALS progression and functional status, placing defects in lipid handling as central to the metabolomic changes observed in ALS (Guo et al., 2025).

In cellular models of ALS astrocytes, reductions in lipid species such as glycerophospholipids, as well as levels of carnitine have been described (Madji Hounoum et al., 2017; Zhu et al., 2025). This disruption to the carnitine shuttle may directly contribute to dyslipidaemia observed in ALS patients. Lipid peroxidation is also observed in astrocytes from human ALS tissues (Shibata et al., 2001), complementing systemic lipidomic cerebrospinal fluid (CSF) signatures of elevated phosphatidylcholine, ceramides and glucosylceramides (Blasco et al., 2017).

Under physiological conditions, astrocytes have a key neuroprotective function in lipid buffering. They can sequester neurotoxic fatty acids (FAs), store them in lipid droplets (LDs), and subsequently process them through the carnitine shuttle for mitochondrial β-oxidation and ketone body production to alleviate neuronal oxidative stress (Liu et al., 2017; Barber and Raben, 2019; Ioannou et al., 2019). This lipid-handling capacity is essential for protecting neurons from lipid-induced oxidative damage. However, in disease, this process becomes maladaptive and LD accumulation becomes excessive with toxic effects. An elevated number of LDs has been observed across SOD1, C9ORF72, FUS and TDP-43 mutant astrocytes in both *in vivo* and *in vitro* human and rodent models, as well as human postmortem tissue (Velebit et al., 2020; Esteve et al., 2026; Marcadet et al., 2026). However, this is not a benign storage, as global lipid accumulation - induced via overexpression of sterol regulatory element-binding protein 2 (SREBP2) - in wildtype mice can induce ALS-like phenotypes including MN death, astrogliosis, diminished body weight, hindlimb paralysis and reduced survival (Dodge et al., 2020). Such effects of LD accumulation could be reduced in human and mouse astrocyte *in vitro* cultures by inhibiting lactate dehydrogenase (LDH) or genetic ablation of LDHA, highlighting the therapeutic potential of modulating of astrocytic metabolism (Esteve et al., 2026). These studies underscore the significance of elucidating the mechanistic underpinnings of lipid dysfunction in ALS, which has so far been understudied.

In addition, astrocytes are the main producers of cholesterol, which is a multifaceted molecule with key roles in the brain including supporting synaptic function, inflammation and cellular membrane integrity (Pfrieger and Ungerer, 2011). Global reductions in SREBP2 transcriptional activity and levels have been described in TDP-43 and SOD1 mouse models and postmortem tissues (Dodge et al., 2020; Egawa et al., 2022). However, the effect of this reduction on astrocytic function in cholesterol homeostasis and synaptic maintenance in an ALS context is yet to described.

### 2.3 Clearance of glutamate

Glutamate is the principal excitatory neurotransmitter in the CNS, but excess extracellular glutamate can lead to neuronal hyperexcitability and excitotoxicity. Astrocytes play a critical role in preventing this through rapid glutamate uptake at the tripartite synapse, primarily via excitatory amino acid transporter 2 (EAAT2/GLT1) (Kim et al., 2011). Once internalised, glutamate can be converted to glutamine for neuronal recycling or enter the tricarboxylic acid (TCA) cycle, linking glutamate handling directly to astrocytic metabolism.

Loss of EAAT2 expression is one of the best characterised astrocytic abnormalities in ALS. Reduced EAAT2 protein levels have been observed in the motor cortex and spinal cord of sALS patients (Rothstein et al., 1995), potentially driven in part by aberrant *EAAT2* mRNA processing (Lin et al., 1998). Similarly, reduced GLT1 expression in SOD1-G93A mouse models accelerates motor decline and reduces survival (Pardo et al., 2006). However, impaired glutamate clearance in ALS may not solely reflect altered transporter expression, but also broader astrocytic energetic dysfunction.

Glutamate uptake is a highly energy-dependent process that relies on intact astrocytic ion gradients, Na^+^/K^+^-ATPase activity, and mitochondrial function. Astrocytic reactivity may further lower the threshold for neuronal hyperexcitability in ALS, including impaired astrocytic Kir4.1 channel function and Na^+^/K^+^-ATPase activity, both of which contribute to maintenance of extracellular ionic homeostasis (Bataveljić et al., 2012). Moreover, transcriptomic analyses across multiple ALS genotypes have identified convergent downregulation of glutamate transport and homeostatic support pathways alongside increased inflammatory and endoplasmic reticulum stress signatures in astrocytes (Ziff et al., 2022). Additionally, as glutamate transport is coupled to sodium influx, astrocytes must expend substantial ATP to restore ionic homeostasis following synaptic activity. Consequently, impaired mitochondrial bioenergetics and reduced metabolic flexibility may compromise the ability of astrocytes to efficiently clear extracellular glutamate.

Together, these findings suggest that impaired glutamate handling in ALS is closely linked to broader astrocytic metabolic dysfunction. Failure of glutamate clearance may therefore represent not only a direct excitotoxic mechanism, but also a downstream consequence of astrocytic bioenergetic collapse.

## 3 Non-cell autonomous effect of impaired astrocytic metabolism on neurons

Substantial evidence supports an important non-cell autonomous contribution of astrocytes to ALS pathogenesis. Astrocytes with ALS mutations can cause MN degeneration regardless of neuronal genotype (Di Giorgio et al., 2008; Marchetto et al., 2008; Haidet-Phillips et al., 2011; Hall et al., 2017), as well as induce physiological deficits (Zhao et al., 2020; Stoklund Dittlau et al., 2023). Although much of the mechanistic understanding of this astrocyte-mediated dysfunction originates from SOD1 models, studies in human stem cell models of C9ORF72-, FUS-, VCP-associated and sALS suggest that impaired astrocytic support and non-cell autonomous neurotoxicity are convergent features across multiple ALS subtypes (Haidet-Phillips et al., 2011; Hall et al., 2017; Zhao et al., 2020; Stoklund Dittlau et al., 2023). Importantly, wildtype astrocytes can support the survival of mutant MNs (Clement et al., 2003).

### 3.1 Loss of metabolic support

Loss of astrocytic bioenergetic functions associated with providing neuroprotective homeostatic support may contribute to MN dysregulation in ALS. Firstly, *in vitro* models exhibit a downregulation of transporters associated with the lactate shuttle pathway in astrocytes expressing SOD1-G93A when in co-culture with wildtype MN, but not monoculture, suggesting neuronal contact is necessary to unmask metabolic dysfunction (Ferraiuolo et al., 2011). Additionally, SOD1-G93A astrocytes could induce changes in mitochondrial function including decreased mitochondrial membrane potential, reduced mitochondrial redox state and increased intra-mitochondrial calcium levels in both wildtype and SOD1-G93A neurons (Bilsland et al., 2008). Recent work in human C9ORF72 induced pluripotent stem cell (iPSC)-derived models has similarly demonstrated astrocyte-dependent alterations in MN mitochondrial function, suggesting that disruption of astrocyte-mediated metabolic support may extend beyond SOD1-linked disease (Stavrou et al., 2025).

In comparison to neurons, astrocytes have a robust antioxidant defence regulated by the Nrf2 pathway (Vargas and Johnson, 2009). Under oxidative stress, Nrf2 dissociates from its inhibitory binder, Keap1, and translocate to the nucleus. There, it drives the expression of enzymes for glutathione synthesis and protective enzymes like SOD, furthering the neuroprotection of astrocytes to MNs. This outsourced antioxidative capacity is vital, as MNs possess limited autonomous glutathione synthesis and are entirely reliant on glial support to neutralise excess polyunsaturated FAs and reactive oxygen species (ROS). However, in ALS, when the astrocytes become chronically reactive, they lose their antioxidant capacity and instead becomes a source of pro-apoptotic signals. For example, glutathione synthetase is downregulated in mutant C9ORF72 iPSC-derived astrocytes (Birger et al., 2019). Clinical markers support this systemic breakdown as alpha-hydroxybutyrate, a specific marker for increased lipid oxidation and oxidative stress linked to glucose-handling issues, is increased in both CSF and plasma of ALS patients (Wuolikainen et al., 2016).

Therefore, the antioxidant and Nrf2 pathway is a compelling target, as decreased expression levels are observed in postmortem tissue of ALS patients (Sarlette et al., 2008) and overexpression of Nrf2 in astrocytes increased glutathione secretion, survival of MNs and delayed onset in both SOD1-G93A mouse model primary cultures and *in vivo* (Vargas et al., 2008; Diaz-Amarilla et al., 2016). Additional evidence supporting enhancement of astrocytic antioxidant capacity demonstrated that overexpression of astrocytic mitochondria-targeted catalase in SOD1-G93A astrocytes could improve their mitochondrial function and revert their toxicity towards MNs (Pehar et al., 2014).

Together, these studies suggest that a loss of astrocytic bioenergetic function support may contribute to MN dysregulation in ALS.

### 3.2 Gain of neurotoxicity

However, research points to a role beyond loss of supportive capacity as concurrently there is a gain of neurotoxicity contributed by dysfunctional astrocytes. Elevated levels of oxidative stress associated genes including *SOD1* and *CHCD10* have also been described in astrocytes from ALS postmortem cortical tissue (O’Neill et al., 2025). Mitochondrial dysfunction and oxidative stress may shift astrocytes from a metabolically supportive state toward a reactive neurotoxic phenotype characterised by secretion of inflammatory cytokines, ROS, and toxic lipid species (Vicente-Gutierrez et al., 2019; Guttenplan et al., 2021). Increased presence of these reactive astrocytes has been observed in the SOD1-G93A mouse model and ALS postmortem tissue, with reduction of these astrocytes extending survival in the mouse model (Liddelow et al., 2017; Guttenplan et al., 2020). Therefore, astrocyte-focused therapies must address both the tendency to lose neuro-supportive roles as well as conversion into neurotoxic astrocytes.

## 4 Therapeutically targeting astrocyte metabolism in ALS

### 4.1 Trialled therapies and their effects on astrocyte bioenergetics

Most preclinical successes have not translated clinically, reflecting two core problems: the over-reliance on SOD1 models which represents a minority of ALS patients and late diagnosis after significant MN loss. Nonetheless, many approved and trialled therapies for ALS have effects on astrocytic function. Riluzole, currently the only drug licenced to treat ALS in the United Kingdom which extends survival by 2–3 months, partially engages astrocyte pathology by enhancing EAAT2-mediated glutamate clearance to inhibit hyperexcitability (Fumagalli et al., 2008). Edaravone, a free radical scavenger approved for ALS and ischemic stroke treatment in the United States, increases Nrf2 expression (Xu et al., 2022), reduces SOD1 deposition in mutant mice and slows MN degeneration (Ito et al., 2008). Although clinical trials have shown slower physical and respiratory function loss than a placebo (Writing Group, Edaravone (MCl-186) ALS 19 Study Group, 2017; Brooks et al., 2022), its effectiveness as a monotherapy is unclear and several key questions about its use remain unanswered (Seok, 2025). Additionally, ceftriaxone was shown to upregulate EAAT2 expression and extend survival in the SOD1-G93A ALS mouse model (Rothstein et al., 2005), but failed to improve functional decline or survival in phase III clinical trials (Cudkowicz et al., 2014).

### 4.2 Astrocytic bioenergetic rescue as a promising therapeutic target

**FIGURE 2:**
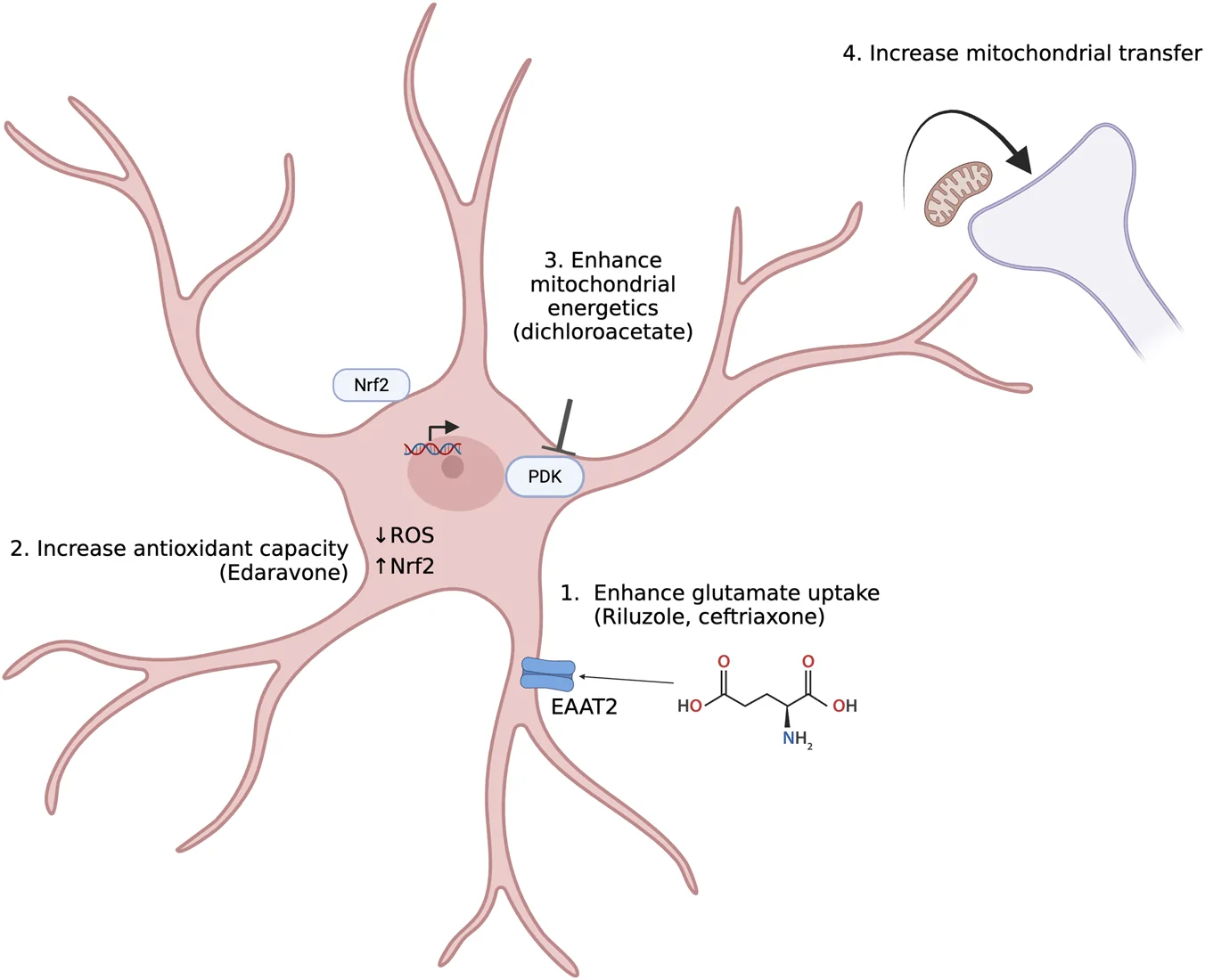
Strategies to therapeutically target dysregulated astrocytic-neuron metabolic coupling in ALS. Therapeutic strategies targeting astrocytic metabolism to enhance neuronal function in ALS are; 1. Enhancement of glutamate uptake; 2. Increasing antioxidant capacity; 3. Enhanced mitochondrial energetics; 4. Transfer of healthy mitochondria from astrocytes to neurons. ALS, amyotrophic lateral sclerosis; EAAT2, excitatory amino acid transporter 2; Nrf2, nuclear factor erythroid 2-related factor 2; ROS, reactive oxygen species. Figure created with BioRender.com.

However, *in vivo* and *in vitro* studies across neurodegenerative conditions suggest that targeting astrocytic metabolism may be a promising therapeutic approach (Le Douce et al., 2020; Birolini et al., 2021; Jiwaji et al., 2022) (Figure 2). In the SOD1-G93A model, dichloroacetate could reduce astrocyte-mediated MN death in culture and extended survival by inhibiting pyruvate dehydrogenase kinases (PDK), which are highly expressed by astrocytes (Miquel et al., 2012; Martínez-Palma et al., 2019). PDK2 knockdown in SOD1-G93A astrocytes was also shown to restore mitochondrial bioenergetic parameters, reduce LD accumulation and improve MN survival, supporting the therapeutic targeting of astrocytic mitochondria in ALS (Miquel et al., 2024).

The transfer of healthy mitochondria from astrocytes to neurons may offer a promising approach to support neuronal metabolism and reduce neuronal degeneration in ALS, as previously demonstrated in models of stroke and Parkinson’s disease (Hayakawa et al., 2016; Cheng et al., 2020). However, it may be difficult to clinically translate this therapy due to the challenges of cellular uptake, enzymatic degradation and delivery (Lan et al., 2026).

## 5 Discussion and future directions

Despite the emerging evidence which supports the idea of progressive failure of astrocyte-mediated metabolic support as a key contributor to ALS pathogenesis, unresolved causality remains. That is whether astrocytic metabolic abnormalities are primary pathogenic drivers, compensatory adaptations or a downstream response to neuronal pathology in ALS. Moreover, this state likely differs across disease stage and ALS subtype, adding further to the complexity. Until recently, studying astrocyte-neuron metabolic crosstalk was methodologically challenging, with emerging platforms including human stem cell models and integrative ‘omic studies enabling promising advancements (Bolaños et al., 2025).

Firstly, advances in two-photon microscopy further allows real-time investigation in living systems, overcoming the static-state limitation of current *in vitro* metabolomics. However, animal models are constrained by interspecies differences (Cáceres et al., 2003). Human organoid and tri-culture approaches provide more physiologically relevant models of multicellular interaction which can be complemented with single cell ‘omics to untangle cell-type specific contributions and clarify cell-subtype-selective susceptibilities. These systems can reveal early contributions to pathogenesis, particularly important for understanding the pre-symptomatic phase of disease when therapeutics are most likely to be effective (Szebényi et al., 2021). When this is paired with CRISPR/Cas9 gene-editing technologies, it allows causality between a given mutation and phenotypes to be ascribed.

Investigating alterations in astrocytic metabolism in ALS cohorts remains challenging as standard FDG-PET lacks cell-type resolution to distinguish astrocyte-specific metabolic contributions from global signals. ^11^C-acetate PET could offer a potential solution for directly assessing astrocytic metabolism as acetate is preferentially metabolised by astrocytes through the TCA cycle rather than neurons and has been utilised in Alzheimer’s disease and multiple sclerosis clinical research (Duong et al., 2021; Kato et al., 2021).

Accounting for heterogeneity remains a key issue. Firstly, astrocyte reactivity in disease is not a binary state but a dynamic continuum with static snapshots from endpoint assays and postmortem study unable to reveal the ongoing complexity. This is further complicated by ALS heterogeneity across genetic subtypes, sites of onset and disease stages. An additional unresolved challenge is capturing regional metabolic heterogeneity, particularly of the spinal cord and motor cortex, and mapping this with disease severity (Hasel et al., 2023), with ongoing efforts utilising spatial transcriptomics to link molecular changes to vulnerable regions in ALS (Gregory et al., 2020).

## 6 Conclusion

ALS pathogenesis may reflect a fundamental failure of astrocyte–neuron metabolic coupling. Bioenergetic collapse drives a cascade of mitochondrial failure, ANLS disruption, and lipid dyshomeostasis that progressively deprives MNs of energy, antioxidant support, and trophic factors. Simultaneously, reactive astrocytes acquire neurotoxic properties that actively accelerate MN death. Many of these mechanisms converge across ALS genotypes and models which argues for astrocyte-targeted strategies. Moreover, emerging human cellular platforms and technological advancements are enabling the resolution of cellular and temporal complexity. Addressing these outstanding questions will require longitudinal, multicellular human models paired with biomarker strategies capable of defining how astrocytic metabolic dysfunction evolves across disease stages and identifying therapeutically tractable windows in ALS.
